# A study of the binary marginal effect of technical barriers to trade on the export of shellfish and aquatic products in China

**DOI:** 10.1371/journal.pone.0324166

**Published:** 2025-09-08

**Authors:** Xiaoming Chen, Xin Shan, Peng Xin, Jian Xu

**Affiliations:** School of Economics and Management, Qingdao Agricultural University, Qingdao, China; Bigelow Laboratory for Ocean Sciences, UNITED STATES OF AMERICA

## Abstract

This study aims to examine the impact of technical barriers to trade (TBT) on China’s shellfish exports, focusing on both the intensive margin (trade volume) and the extensive margin (trade type). The research includes shellfish and aquatic products such as scallops, mussels, clams, oysters, and abalone, using HS-6 codes from 2003 to 2020. Panel data is employed for analysis. The findings reveal that TBT notifications positively influence the extensive margin by increasing export types but have a less significant effect on the intensive margin. Economic scale, variable trade cost, and productivity level affect both margins. To mitigate the effects of TBT, China should diversify its shellfish exports. Additionally, reducing trade costs can further enhance China’s shellfish export competitiveness.

## 1. Introduction

According to the *China Fishery Statistical Yearbook*, China has been a major producer and exporter of aquatic products since 1989. The total output of aquatic products has ranked first in the world for more than 30 years, and the total export volume has ranked first in the domestic export volume of bulk agricultural products for 13 consecutive years (2002–2015). In 2012, the trade surplus reached USD 10.985 billion, exceeding USD 10 billion for the first time. As of 2022, the export value of aquatic products was USD 23.01 billion, ranking first in the world. Aquatic products have become one of China’s most advantageous agricultural products and greatly promote the development of China’s foreign trade [1].

With the expansion of the export market of China’s aquatic products, there are more and more incidents involving the quality and safety of shellfish and the related plant and animal diseases due to the biological characteristics of selective filter feeding in shellfish [2]. Among them, the most influential one was the “Edible Shellfish Poisoning Incident” that occurred in the mid-1980s when China exported to the European Union (EU). The exposure of this incident caused the EU to completely ban Chinese bivalves from 1997 to March 4, 2016. This ban for nearly 20 years has had a serious impact on the export of bivalve mollusks from China. The EU and other western countries strengthened their strict control over the quality and safety of imported aquatic products, and the import standards have been raised repeatedly [3]. Therefore, in order to ensure the quality of imported products, technical barriers to trade (TBT) have gradually become the main means to replace traditional non-tariff barriers because of their advantages in terms of facilitation and concealment [4,5], especially since the turn of the 21st century. At present, the most commonly used trade protections in the world are the Agreement on the Application of Sanitary and Phytosanitary (SPS) Measures and the TBT. At first, the fundamental purpose of the SPS measures was to protect the safety and health of human beings, animals, and plants by disclosing product quality information and through other effective means [6]. However, many countries, especially developed countries, have gradually overused this agreement or even used it as a means of restricting imports, disrupting market order and raising the threshold of exporting countries, which has brought export pressure to many countries [7]. Moreover, due to differences in technological level and resource endowment, the SPS measures have gradually become the main obstacle for developing countries wishing to export to developed countries [8,9].

Regarding the impact of TBT, scholars at home and abroad mainly analyze the concept of “dual margin”, and there are many studies on the export of agricultural products that use this concept [10]. The main method is through the gravity model, taking SPS/TBT measures as explanatory variables, together with variables such as gross domestic product (GDP), distance, and population, to measure the degree of influence of TBT on the intensive and extensive margins of agricultural exports. At present, this impact is mainly divided into two types, and many studies focus on restrictions. For example, Huang and Cheng [11] stated that SPS measures produce a prohibition effect equivalent to tariffs, which is mainly manifested in the fact that SPS measures increase trade costs, thus reducing the international competitiveness of products. Crivelli and Groeschl [12], using the Heckman selection model, also reached a similar conclusion; they stated that strict SPS measures will reduce the possibility of exports, but once they enter the destination country, they will increase the trade flow. The research of Zhang and Zhu [13] not only verified the negative impact identified by the above-mentioned scholars but also concretized the inhibitory effect. They argued that the negative impact is mainly reflected in the suppression of the quantity and in the increase in export costs. The former is mainly reflected in the intensive marginal (IM) of exports in developed countries [14], while the extent of the latter in developing countries mainly depends on the export standards set by developed countries. The stricter the standards, the higher the production costs of developing countries [15]. In addition, many scholars also found that TBT will promote the development of export trade. For example, Peng [16] found that through the establishment of a gravity model the TBT in developed countries significantly increase the quantity of China’s exports to trading partners. Furthermore, this promotion effect is more significant in the types of aquatic product exports. Yang and Wu [17] and Guo [18] found that the growth in China’s total aquatic product exports is mainly attributable to the extensive margin (EM), which not only increases the export volume but also accompanies the improvement of China’s international status.

From this literature review, it seems that after positive and negative impacts occur, developed and developing countries actually adopt two completely different coping strategies. For developed countries, their abundant capital and high-precision technology often encourage them to develop more new products that meet the standards through innovative new technologies, and they rely on the restrictions of barriers to turn disadvantages into advantages, which has a negative effect on export enterprises [19]. In this way, exports are promoted by expanding the margin [20]. Meanwhile, developing countries tend to adopt a conservative low-price competition strategy, and they continue to choose traditional markets or switch to other new markets with low standard requirements and promote the development of overall trade through the IM [21].

Therefore, in order to study the influence of TBT in developed countries on China’s export trade, this study uses the data of shellfish exports in the 18-year period from 2003 to 2020 and draws lessons from the theory of enterprise heterogeneity put forward by Melitz [22] and Helpman et al. [23] to explain the differences in trade flows among countries through the differences in productivity among enterprises. Based on this, a binary marginal model is constructed, the influencing factors are empirically analyzed from the perspectives of the IM and the EM, and corresponding countermeasures and suggestions are put forward.

The study makes several contributions to the existing literature. Firstly, it employs a binary marginal model to analyze the trade data of shellfish and aquatic products, offering a nuanced understanding of how TBT affects both the intensive and expansion margins of exports. This analytical approach enables a more comprehensive examination of the effect of TBT on trade, compared to previous studies that often focused solely on one aspect of the margin. Secondly, this study extends the binary marginal theoretical model by incorporating explanatory variables such as technical barriers and dummy variables representing economic crises. This allows for a more accurate assessment of the impact of TBT on shellfish exports, while also considering other factors that may influence trade flows. By doing so, the study provides valuable insights into the complexities of international trade and the role of non-tariff barriers in shaping trade patterns. Lastly, the current study offers practical recommendations for policymakers and exporters on how to navigate the challenges posed by TBT. The study is organized as follows. Section 2 presents the literature review, Section 3 presents the methodology, Section 4 shows the results, and Section 5 discusses the results. Finally, Section 6 concludes.

## 2. Literature review

The export of shellfish and aquatic products in China is a topic of interest in the realm of international trade dynamics [3]. Several studies have delved into the impact of various non-tariff measures, including TBT, on the export of agricultural products, including aquatic products. World Trade Report [24] focused on the trade effects of non-tariff measures, including SPS and TBT measures, on Chinese exports of fresh vegetables, fish, and aquatic products during the period between 1992 and 2004. This research highlights the significance of regulatory barriers in shaping export trends and market access for Chinese aquatic products. Moreover, the impact of technical barriers on international trade has been a subject of investigation in the literature. OECD [25] explored the effects of technical barriers on US agricultural exports, emphasizing the significance of regulations in influencing trade outcomes. This suggests that technical barriers, including TBT, can have a substantial impact on the export of agri-food products, such as shellfish and aquatic products. In the context of aquatic products, Wang et al. [26] analyzed the growth dynamics of aquatic products exported from China and Vietnam using the ternary marginal method. Their study provides insights into the trade growth patterns and sustainability of aquatic product exports in the region. Wei et al. [27] pointed out that exporters can withstand TBT shocks by optimizing the reallocation of resources within their firms across different markets and products. In addition, studies have focused on the binary margin of trade, analyzing both the IM (trade volume) and the EM (trade type). Chaney [28] provided foundational insights into this framework, while the research in question draws on this methodology to study the impact of TBT on China’s shellfish exports. It uses notified quantities of products as a proxy to reflect the degree of impact and employs econometric models to examine the relationship between TBT and shellfish exports. Chen and Bao [29], using firm-level data, found that TBT decrease both the extensive and intensive margins of small firms. Overall, the literature review indicates a growing interest in understanding the binary marginal effect of TBT on the export of shellfish and aquatic products in China, which can help researchers gain valuable insights into the factors influencing market access and competitiveness in the global trade landscape.

## 3. Methodology

### 3.1. Sample

According to Sun and Niu [30], scallops, mussels, clams, oysters, and abalone belong to the class of shellfish and aquatic products. Taking all these into consideration, the HS codes for the shellfish in this study included 030710, 030711, 030712, 030719, 030721, 030722, 030729, 030731, 030732, 030739, 030771, 030772, 030779, 030781, 030783, 030787, 030789, 160551, 160552, 160553, 160556, and 160557. In addition, in the selection of countries, China’s main trading partners and countries with high trade barriers were the first choices. In this study, the United States, Canada, Japan, and South Korea were selected.

### 3.2. Measurement of TBT

TBT as the most effective non-tariff barriers are difficult to measure [31]. This has become a key factor affecting the export of aquatic products in China and is also the main obstacle to the export of agricultural products in China to developed countries [32,33]. This study draws on the measurement method of Bao and Zhu [34] and takes both TBT and SPS measures into consideration, in order to obtain complete measurement results.

Regarding the measurement of TBT/SPS, the more commonly used methods are the following: the stock index method, tariff equivalence method, calculation of the trade barrier coefficient, quantity of special trade concerns, etc. Among them, the most used method is the stock index method [35]. It mainly measures the impact and degree of China’s trade according to the number of notifications from the World Trade Organization (WTO). The departments are analyzed and compared; then, the sharing of information resources is realized [36]. The second most used are the frequency ratio and the trade coverage ratio, which are more specific than the stock index method and can be refined to the frequency of the occurrence of barriers in a certain sector of a certain company in a certain country, so as to correctly assess the scope and degree of trade impact. The disadvantage is that the data and information cannot be fully obtained, and the amount that needs to be calculated is too large; thus, they are only suitable for small-scale research. Finally, a small number of studies choose to use standardized quantitative indicators as the quantitative basis. For example, some scholars measure the quality and safety of products by calculating the maximum residue level of drugs. Export trade is mainly restricted by the food quality standards of developed countries [37].

Based on the above analysis, in terms of the selection of measurement indicators, and in view of the availability of data and the convenience of the empirical analysis below, this study used the notified quantity of products to reflect the degree of the impact of TBT on shellfish and aquatic products.

### 3.3. Calculation of intensive and extensive margins

The analysis of the binary margin in this study draws on the measurement method proposed by Hummels and Klenow [38] and studies from the perspectives of the IM (trade volume) and the EM (trade type). Therefore, the binary margin is defined as follows:


$${\text{IM}}_{\text{ijt}}=\frac{\sum_{\text{k}\in {\text{K}}_{\text{ijt}}}{\text{P}}_{\text{ijk}}{\text{X}}_{\text{ijk}}}{\sum_{\text{k}\in {\text{K}}_{\text{ijt}}}{\text{P}}_{\text{wjk}}{\text{X}}_{\text{wjk}}}$$ (1)

$${\text{EM}}_{\text{ijt}}=\frac{\sum_{\text{k}\in {\text{K}}_{\text{ij}}}{\text{P}}_{\text{wjk}}{\text{X}}_{\text{wjk}}}{\sum_{\text{k}\in {\text{K}}_{\text{wj}}}{\text{P}}_{\text{wjk}}{\text{X}}_{\text{wjk}}}$$ (2)

In Equations (1) and (2), i and j represent the exporting country (China) and the importing country, respectively; w represents the reference country (generally, the world is selected as the reference); k is the export type of shellfish and aquatic products; and K_ij_ is the shellfish that China exports to country j. Regarding the collection of aquatic product export types, K_wj_ represents the collection of shellfish and aquatic products exported by the world to country j; P_ijk_ indicates the price of China’s export of shellfish and aquatic products to country j; P_wjk_ indicates the price of shellfish and aquatic products exported by world w to country j; X_ijk_ indicates the quantity of shellfish and aquatic products exported by China to country j; X_wjk_ indicates the number of shellfish and aquatic products exported from world w to country j.

IM refers to the ratio of China’s export of shellfish and aquatic products to country j to the world’s trade volume of shellfish and aquatic products exported to country j with the same type of shellfish and aquatic products as China. The greater the proportion of shellfish and aquatic product exports, the greater the margin.

EM refers to the ratio of the world trade volume of shellfish and aquatic products exported to country j with the same type of shellfish and aquatic products exported to China to the world trade volume of all types of shellfish and aquatic products exported to country j. The more diverse the types of shellfish a country exports, the more likely they are to be exported.

According to the above calculation, the binary margins of China’s export changes to Japan, the United States, South Korea, and Canada from 2003 to 2020 were calculated. The specific data are shown in Table 1.

**Table 1:** Binary marginal of China’s shellfish and aquatic products.

Year	United States		Canada		Japan		South Korea	
	IM	EM	IM	EM	IM	EM	IM	EM
2003	0.002	0.305	0.004	0.201	0.276	0.856	0.510	0.568
2004	0.091	1.000	0.097	0.574	0.304	1.000	0.308	1.000
2005	0.186	0.923	0.110	0.754	0.322	1.000	0.316	1.000
2006	0.171	0.922	0.123	0.883	0.140	1.000	0.462	1.000
2007	0.163	0.915	0.072	0.814	0.143	1.000	0.236	1.000
2008	0.154	0.853	0.107	0.747	0.094	1.000	0.335	0.995
2009	0.201	0.706	0.174	0.362	0.127	1.000	0.409	0.987
2010	0.477	0.588	0.296	0.397	0.128	0.827	0.561	0.985
2011	0.445	0.492	0.313	0.395	0.207	0.999	0.668	1.000
2012	0.474	0.571	0.290	0.658	0.495	1.000	0.836	0.992
2013	0.437	0.632	0.295	0.654	0.562	0.948	0.852	0.997
2014	0.527	0.614	0.302	0.699	0.559	0.942	0.869	0.997
2015	0.580	0.662	0.299	0.793	0.597	0.880	0.877	1.000
2016	0.493	0.769	0.322	0.849	0.558	0.935	0.872	0.997
2017	0.504	0.723	0.388	0.700	0.574	0.943	0.857	0.948
2018	0.529	0.708	0.361	0.832	0.601	0.936	0.866	0.955
2019	0.367	0.673	0.352	0.789	0.585	0.919	0.794	0.997
2020	0.332	0.732	0.411	0.821	0.540	0.992	0.795	0.927

According to Table 1, from the perspective of EM, the average extensive margins of China’s shellfish and aquatic products exported to Japan and South Korea are 0.954 and 0.964, respectively, both of which are above 0.95; during 2004–2008, there was even a phenomenon where the EM was 1. This shows that China’s shellfish exports to Japan and South Korea have the largest variety and are basically at the world level. Furthermore, the types of exports change little every year, making these countries stable and lasting trading partners for China. In addition, the United States and Canada have average extensive margins of 0.7 and 0.662, which are lower than those of Japan and South Korea. Although the types of exports are not so rich, the growth rate is very large. Among them, Canada’s EM in 2020 increased by 308.5% compared with that in 2003, making it the fastest growing country among the four countries, especially in the 2008 financial crisis. After that, from 2009 to 2020, it had a state of steady growth, and it belongs to the group of trading partners with huge potential. From the perspective of IM, the average IM of China’s shellfish exports to South Korea is 0.635, and it ranks first among the four countries. Combined with the EM, South Korea is the country with the largest number and the richest variety of shellfish and aquatic products exported by China, while Japan, which is also a country with an EM greater than 0.9, has an IM of only 0.378. The reason for this may be that Japan is the country with the strictest trade barriers to China. In the 20 years from 2003 to 2022, Japan notified China that there were 193 batches of unqualified shellfish products; in this respect, it ranked first among the four countries. Therefore, the quality and safety of China’s export products can hardly meet the standards set by the Japan’s Ministry of Health, Labour and Welfare, and the export volume is naturally much lower than that to South Korea, which has fewer barriers. Unlike the EM, the IM growth of the United States and Canada is relatively stable, especially from 2012 to 2018, which indicates that the trade exchanges between China and the United States are relatively close, and the United States and Canada have gradually established a good situation of complementary trade that represents win-win cooperation with China. However, after the Sino-US trade war in 2018, the IM of China’s exports to the United States dropped significantly, and those to Japan and South Korea both declined slightly. This shows that this trade war had a significant hindering effect on the export of shellfish from China and serves to warn China’s shellfish and aquatic product-related export enterprises to enrich the types of shellfish products and make good use of opportunities of the Belt and Road Initiative. While stabilizing traditional markets, the Chinese government need to actively explore emerging markets such as Southeast Asia, reduce trade risks, and improve international competitiveness.

### 3.4. Variables and models

An authoritative analysis framework of the binary margin influencing factors was proposed by Chaney [28], and the theoretical model of the binary margin is constructed as follows:


$$\begin{array}{c} {𝐞}_{𝐢𝐣}{=\lambda }_{3}({\frac{{𝐘}_{𝐣}}{𝐘})}^{\mathrm{\backslash nofrac}\left(\sigma -1\right)/\gamma }({\frac{\theta_{𝐣}}{\tau_{𝐢𝐣}})}^{\sigma -1}({\frac{\varphi }{{𝐰}_{𝐢}})}^{\sigma -1},\varphi \geq \varphi_{𝐢𝐣} \end{array}$$ (3)

$$\begin{array}{c} {\text{N}}_{\text{ij}}=({\frac{\sigma }{\sigma -1})}^{\sigma -1}\frac{{\text{Y}}_{\text{i}}{\text{Y}}_{\text{j}}}{\text{Y}}{\text{f}}_{\text{ij}}^{\mathrm{\backslash nofrac}-\gamma /\left(\sigma -1\right)}({\frac{{\text{w}}_{\text{i}}\tau_{\text{ij}}}{\theta_{\text{j}}})}^{-\gamma } \end{array}$$ (4)

In Equations (3) and (4), e_ij_ represents the IM, and N_ij_ represents the EM; λ_3_ is a constant; ф and φ_ij_ represent the productivity and the threshold of the enterprise, respectively. Only when the productivity is greater than or equal to the threshold will the enterprise choose to export. $\frac{Y_{i}Y_{j}}{Y}$ represents the economic scale; w_i_ represents the labor productivity; τ_ij_ andf_ij_ represent the variable trade cost and the fixed trade cost; θ_j_ represents the multilateral trade resistance between trading countries and other countries, except for China; σ is the substitution elasticity of the products; and γ is a parameter of firm heterogeneity. The smaller the value of γ, the stronger the firm heterogeneity.

It can be seen that the influencing factors of the binary margin are mainly composed of the production scale, productivity, variable and fixed trade costs, and multilateral trade resistance. The influencing factors of the IM and the EM are slightly different; this difference is mainly reflected in the fact that the fixed trade cost only affects the EM and has no effect on the IM. In order to examine the impact of the TBT, this study expands the binary marginal theoretical model and adds the explanatory variable TECH to represent the barrier and the dummy variable SHOCK to represent the economic crisis. Therefore, the models are constructed as follows:


$$𝐥𝐧\;𝐈𝐌=\;\alpha_{0}\;+\;\alpha_{1}\;𝐥𝐧\;\left({𝐓𝐄𝐂𝐇}_{𝐭-1}\right)\;+\;\alpha_{2}\;𝐥𝐧\;𝐒𝐂𝐀𝐋𝐄\;+\;\alpha_{3}\;𝐥𝐧\;𝐃𝐈𝐒𝐓\;+\;\alpha_{4}\;𝐥𝐧\;𝐏𝐑𝐎𝐃\;+\;\alpha_{5}\;𝐥𝐧\;𝐌𝐑𝐄𝐒\;+\;\alpha_{6}\;𝐒𝐇𝐎𝐂𝐊\;+\;\mu$$ (5)

$$𝐥𝐧\;𝐄𝐌=\;\beta_{0}\;+\;\beta_{1}\;𝐥𝐧\;\left({𝐓𝐄𝐂𝐇}_{𝐭-1}\right)\;+\;\beta_{2}\;𝐥𝐧\;𝐒𝐂𝐀𝐋𝐄\;+\;\beta_{3}\;𝐥𝐧\;𝐃𝐈𝐒𝐓\;+\;\beta_{4}\;𝐥𝐧\;𝐏𝐑𝐎𝐃\;+\;\beta_{5}\;𝐥𝐧\;𝐅𝐑𝐄𝐄\;+\;\beta_{6}\;𝐥𝐧\;𝐌𝐑𝐄𝐒\;+\;\beta_{7}\;𝐒𝐇𝐎𝐂𝐊\;+\;\varepsilon$$ (6)

In Equations (5) and (6), IM and EM represent the intensive margin and the expansion margin, respectively, and the results calculated with Equations (1) and (2) are substituted into the logarithm. The former represents China’s export volume of shellfish products to trading countries, and the latter represents the possibility of China’s export of shellfish products to trading countries. α and β are the estimated parameters; μ and ε are the error terms; and their respective variables are defined as follows:

(1) TECH represents the TBT, which is a core explanatory variable. In the inquiries of websites such as the China Technical Trade Measures Network (http://www.tbtsps.cn/) and WTO/TBT-SPS National Notification and Enquiry of China (http://www.tbt-sps.gov.cn/), the data on the detention/recall of shellfish products in China are compiled, to measure the impact of TBT on shellfish exports. As the notification not only has an impact on the current year but may also cause a delay in time, this study uses the number of notifications with a lag of one year. Generally speaking, the more barriers that shellfish products suffer, the lower the export volume; so, the expected impact on the binary marginal is negative.
(2) SCALE as an explanatory variable represents the economic scale. It is expressed as the relative agricultural added value, that is, the ratio of the agricultural added value of trading countries to that of China. The larger the market size, the stronger the demand capacity of the importing country; so, the expected impact on the binary margin is positive. The data come from the World Bank (WDI) database (https://databank.worldbank.org).
(3) DIST represents variable trade cost, which is an explanatory variable. As with all fresh agricultural products, the transportation of shellfish and aquatic products is greatly affected by time constraints [39]. Therefore, this study draws on the research method of Helpman et al. [23] and Peng et al. [40] and uses the distance between the capitals of China and its trading partners as an effective measure with which to measure the variable trade cost of exports. Generally speaking, the further the distance between the two countries, the longer the transportation time, the higher the cost of transportation and the cold chain; so, the expected impact on the binary margin is negative. The data come from the CEPII database (http://www.cepii.fr/).
(4) PROD represents productivity level, which is an explanatory variable. In this study, the ratio of the per capita agricultural added value of the importing countries to that of China is used as a measure of the labor productivity level of the agricultural practitioners. According to Melitz [22], the higher the productivity of a trading country, the more likely it is to export. Therefore, the demand capacity of aquatic products in China is correspondingly reduced, which leads to the reduction in the quantity and variety, resulting in the reduction in the binary margin. Therefore, the expected impact is negative. The data come from the World Bank (WDI) database.
(5) FREE represents fixed trade cost, which is an explanatory variable. This study uses the calculation method of Tian and Du [41] and draws on the formula proposed by Head [42] to measure the size of fixed trade costs. The formula is as follows:

$${𝐅𝐑𝐄𝐄}_{𝐢𝐣𝐭}=\sqrt{\frac{{𝐄}_{𝐢𝐣𝐭}*{𝐄}_{𝐣𝐢𝐭}}{{𝐄}_{𝐢𝐢𝐭}*{𝐄}_{𝐣𝐣𝐭}}}$$ (7)

In Equation (7), i represents the exporting country (i.e., China), and j represents the four countries of the United States, Canada, Japan, and South Korea as the export destination countries. E_ijt_ is the trade volume of shellfish products exported from China to the destination country during the t period, and the same is true for E_jit_; E_iit_ and E_jjt_ are the total domestic sales of China and the destination country in period t, respectively, and their values are approximated by subtracting the total exports from the GDP of each country. In order to avoid errors caused by the formula calculation, this study standardized the FREE. Generally speaking, the higher the degree of trade freedom, the lower the cost of entry and the more conducive to trade with China; so, the expected impact is positive. The data come from the United Nations Commodity Trade database.

(6) MRES represents multilateral trade resistance, which is an explanatory variable that measures the trade resistance of countries other than China to China’s trading partners. Generally speaking, the larger the value, the greater the resistance, and the more likely the country is to conduct trade with China; so, the expected impact is positive. Guided by Shao et al. [43] and Sang and Wang [44], the authors of this study believe that there is no symmetrical trade cost (ø_ij_ ≠ ø_ji_) between the two countries and draw lessons from the formula derived by Head [42] and Novy [45], which is as follows:

$$𝐌𝐑𝐄𝐒=({\frac{{𝐱}_{𝐣𝐣}/{𝐲}_{𝐣}}{{𝐲}_{𝐣}/{𝐲}^{𝐰}})}^{\frac{1}{\sigma -1}}{𝐭}_{𝐣𝐣}$$ (8)

In Equation (8), x_jj_ represents the domestic sales of the destination country, and the calculation method is the same as that given above. It is approximated by subtracting the total export volume from the GDP of that country. y_j_ and y^w^ represent the GDP of the export destination country and the GDP of all the countries in the world, respectively, and t_jj_ is the economic cost of domestic trade. This study also uses the method of Tian and Du [41], measures it using the trade freedom index of the export destination country, and makes a logarithm and a standardization of this index. As for the value of σ, using the calculation of the trade cost of aquatic products in the study of Shao et al. [43], it was selected as the substitution elasticity of aquatic products. The export data were obtained from the United Nations Commodity Trade database, the GDP was obtained from the World Bank database (all the data are in current US dollars), and the trade freedom index was obtained from the Economic Freedom Index report.

(7) SHOCK represents external shock, which is an explanatory variable. The economic shock in the international environment will have a certain degree of impact on international trade [46]. In the panel data from 2003 to 2020, in addition to the impact of the 2018 financial crisis, the impact of the COVID-19 pandemic in 2020 is also very significant [47]. Therefore, this study considered the lag of the economic crisis by assigning a value of 1 to four years (i.e., 2008, 2009, 2019, and 2020) and a value of 0 to the rest of the years. The expected impact is negative.

To sum up, all variables except for the dummy variable are logarithmic, and the dollar measurement is calculated at the current price. This purpose is to unify the units and reduce the impact of error terms.

## 4. Results

### 4.1. Unit root test

Using Stata 17, before the mixed regression of the model, in order to strengthen the stationarity of the panel data series, the Phillip Perron (P.P.) unit root test was first performed on each variable. Because of the time trend item in the model, this study tested the dependent variables (IM and EM) and the core explanatory variables (TECH and SCALE), which are greatly affected by the time trend item. It was found that the *p* value of SCALE was not significant at the 5% level after the test, indicating that the first-order difference was needed to stabilize the original data series. The difference results are shown in Table 2.

**Table 2:** Unit root test.

Variable	Statistics	*p* value
IM	20.7909	0.0000
EM	27.5561	0.0000
TECH	8.5397	0.0000
SCALE	2.6810	0.0037

Notes: *p* values are based on chi-square test.

### 4.2. Descriptive statistics

Table 3 showed the descriptive statistics of each variable. In order to avoid the multi-collinearity issue, the variance inflation factor (VIF) test was first carried out. The results in Table 4 showed that the VIF values of each variable in the IM and the EM were less than 4, so there was no multi-collinearity problem. Then, the autocorrelation test was carried out, and it was found that the p values were 0.045 and 0.01, respectively. Both of them rejected the null hypothesis at the 5% level, and there was no autocorrelation; thus, the relative robustness of the model was determined.

**Table 3:** Descriptive statistics.

Variable	N	Mean	Standard deviation	Minimum	Maximum
IM	72	−1.212	1.026	−6.215	−0.131
EM	72	−0.235	0.313	−1.604	0
TECH	72	1.143	1.119	0	3.497
SCALE	68	0.041	0.610	−0.427	3.508
DIST	72	8.272	1.061	6.862	9.320
PROD	72	1.316	0.622	0	2.396
FREE	72	0.597	0.300	0	1
MRES	72	0.902	0.522	0	1.746
SHOCK	72	0.222	0.419	0	1

**Table 4:** VIF test.

Variable	IM	EM
TECH	1.05	1.06
SCALE	1.36	1.36
DIS	1.08	1.08
PROD	1.35	2.45
FREE	–	2.18
MRES	1.07	1.07
Mean VIF	1.19	1.56

Notes: “–” indicates that these variables were excluded in regression models.

### 4.3. Empirical results

According to the above test, the data series at this stage is in a relatively stable state, and on this basis, the regression analysis can be carried out. Tables 5 and 6 presented the regression results. Because this study dealt with panel data, the Hausman test was performed first to determine whether a fixed effects model or a random effects model should be adopted. The results showed that the null hypothesis cannot be rejected at the 1% level; that is, a random effects model should be used. Therefore, columns (1), (2), and (3) in Tables 5 and 6 were the results of random effects regression; among these, SHOCK was added in column (2), and the year fixed effect was added, mainly because the impact of the economic crisis on all the countries was the same. On the basis of column (3), the individual fixed effects were added. In addition, regarding the data, there were many zeros in the IM and EM of the explained variables, which were limited data. Although we carried out logarithmic processing in the model, ordinary least squares (OLS) could not achieve a better regression effect. Therefore, guided by Qian and Xiong [48], the Tobit model was used to set the left limited data. The interval was estimated as a robustness test, and the year and individual fixed effects were constrained; the results were presented in columns (4) and (5) of Tables 5 and 6.

**Table 5:** Regression results (Dependent variable: IM).

Variable	(1)	(2)	(3)	(4)	(5)
TECH	0.001 (0.039)	−0.003 (0.034)	0.0003 (0.020)	0.002 (0.028)	0.0003 (0.030)
SCALE	−0.269*** (0.080)	−0.183*** (0.030)	−0.183*** (0.045)	−0.183*** (0.060)	−0.183*** (0.062)
DIST	−0.273*** (0.042)	−0.271*** (0.036)	−0.273*** (0.008)	−0.272*** (0.030)	−0.273*** (0.032)
PROD	−0.924*** (0.082)	−0.990*** (0.080)	−0.999*** (0.067)	−0.992*** (0.071)	−0.999*** (0.063)
MRES	−0.193** (0.084)	−0.112** (0.053)	−0.126 (0.102)	−0.114* (0.062)	−0.126* (0.065)
SHOCK	–	−0.520*** (0.087)	−0.521*** (0.039)	−0.520*** (0.098)	−0.521*** (0.084)
Constant	2.534*** (0.374)	2.648*** (0.247)	2.684*** (0.243)	2.655*** (0.271)	2.684*** (0.286)
Year fixed effects	No	Yes	No	Yes	No
Individual fixed effects	No	No	Yes	No	Yes
*R* ^2^	0.746	0.838	0.838	–	–
Prob>*χ*^2^	0.0000	0.0000	–	0.0000	0.0000
Wald	182.08	1316.97	–	305.06	352.95

Notes: “–” indicates that these variables were excluded in regression models, or unobservable; * *p* < 0.10, ** *p* < 0.05, *** *p* < 0.01; standard errors are in parentheses.

**Table 6:** Regression results (Dependent variable: EM).

Variable	(1)	(2)	(3)	(4)	(5)
TECH	0.040** (0.020)	0.040** (0.017)	0.042*** (0.009)	0.040** (0.018)	0.042** (0.018)
SCALE	0.052 (0.040)	0.036** (0.017)	0.042* (0.024)	0.039** (0.037)	0.042* (0.038)
DIST	−0.170*** (0.021)	−0.169*** (0.026)	−0.170*** (0.01)	−0.169*** (0.019)	−0.170*** (0.019)
PROD	0.083* (0.049)	0.101*** (0.029)	0.116** (0.056)	0.109** (0.055)	0.116** (0.052)
FREE	0.133 (0.085)	0.205 (0.126)	0.200 (0.22)	0.201 (0.099)	0.200 (0.096)
MRES	−0.037 (0.042)	−0.037 (0.053)	−0.045 (0.089)	−0.041 (0.039)	−0.045 (0.04)
SHOCK	–	0.073 (0.075)	0.078 (0.065)	0.075 (0.067)	0.078 (0.062)
Constant	1.008*** (0.207)	0.915*** (0.157)	0.915*** (0.156)	0.914*** (0.200)	0.915*** (0.207)
Year fixed effects	No	Yes	No	Yes	No
Individual fixed effects	No	No	Yes	No	Yes
*R* ^2^	0.535	0.544	0.545	–	–
Prob>*χ*^2^	0.0000	0.0000	–	0.0000	0.0000
Wald	70.10	112.28	–	88.25	81.55

Notes: “–” indicates that these variables were excluded in regression models, or unobservable; * *p* < 0.10, ** *p* < 0.05, *** *p* < 0.01; standard errors are in parentheses.

## 5. Discussion

As shown in Tables 5 and 6, according to the regression results in column (1), TECH has a significant positive impact on only the EM. Specifically, when the number of TBT notifications of the trade target country in the previous period increases by 1 unit, the export types increase by 4%. The reason for this may be that the export of China’s shellfish products is mainly based on fresh and live primary products, and the strict barrier measures have relatively few restrictions related to these products. Compared with other exporting countries, China adopts low-price competition strategies or transfers target markets and exports products that do not meet the new standards to Southeast Asia and other countries with relatively loose barriers, so that these products have a relatively weak competitive advantage in a short period of time, and a small increase in export volume occurs [49]. In addition, China has deeply realized the insufficiency in the deep processing of shellfish products for export and is increasing capital investment in order to continue improving science and technology research and development (R&D) [50]. The strict foreign TBT in foreign countries will also have a similar negative effect to those in developed countries. Through the development of new products to expand the types of export products, the total export volume will increase, and, at the same time, the risk to China’s trade presented by the market will be reduced. Therefore, the increasing effect of TBT on China’s shellfish exports is mainly driven by the EM.

Economic scale (SCALE) has a significant positive correlation with the EM and a significant negative correlation with the IM, which is inconsistent with the expectation. This shows that the increase in the economic scale of trading partner countries promotes growth in the export types of shellfish products from China, but it has a significant inhibitory effect on the products that have already been exported. A possible reason might be that most of the products that China has exported to trading partners are primary products. With the increase in the economic scale of trading partners, the per capita income will also increase, the consumption level of residents will rise, and the requirements for the quality of shellfish products will become higher and higher; that is, shellfish products and their processed products that are of a higher quality will be pursued, and this will prompt China to develop more new products to meet the needs of importing countries. Thus, it will play a more important role in promoting the EM.

The results for variable trade cost (DIST) is consistent with previous studies [23,48], indicating that using the distance between the capitals of two countries to measure the DIST is robust. That is, the longer the distance between the two countries, the higher the transportation cost. For fresh shellfish products, the time and environmental requirements are very strict, and a complete supply chain system and strong cold chain equipment are required to complete the distribution [51]; this will undoubtedly increase the cost for the exporting country, reducing the export enthusiasm of the exporting country’s enterprises. Therefore, it has a significant negative impact on both the IM and the EM, and the negative impact on the IM is greater.

Productivity level (PROD) has a significant negative effect on the IM and a positive effect on the EM. The higher the productivity of the trading partner country, the stronger the supply capacity, indicating that the greater the possibility of export, the higher the probability of export. In contrast, the demand for China decreases accordingly, so China’s export volume is negatively correlated with the productivity of its trading partners. In addition, shellfish products are mostly labor-intensive products [52], which can solve problems relating to employment; an increase in productivity will cause the export types of aquatic products to become capital-intensive. The types of exports will gradually be enriched, and the social division of labor will become more specialized. As a result, the trading partners will increase China’s requirements for the diversification of shellfish export types accordingly and raise the barriers to entry into the market. Therefore, there will be a small increase in the EM of China’s shellfish products, but the effect of this increase is far less than the constraint effect brought about by the reduction in demand. The possible reason for this is that the current employees in the shellfish industry in China are mostly rural laborers. In general, their ability to accept new products and new technologies is low, and there is a lack of professional scientific research talents.

FREE has a positive effect on the EM. This shows that the higher the degree of economic freedom, the stronger the degree of market facilitation and the lower the cost and risk of entering the market. Therefore, it is more conducive to the development of trade between countries. A higher degree of economic freedom of trading partner countries will promote the optimization of China’s shellfish product export structure, which is conducive to exporting new products and new types.

The impact of MRES on both the IM and the EM is negative, while this impact on the EM is not significant, which is different from Qian and Xiong [48] and Anderson‚and van Wincoop [53]. It is mainly because of the different measurement methods of multilateral resistance. Most scholars learn from Kancs [54], assuming that there are symmetric trade costs between the two countries, and they make calculations based on this premise. It is believed that the greater the trade resistance that a trading partner country faces from countries other than China, the easier it will be to conduct trade with China. However, this assumption is unrealistic. Many studies have shown that there is heterogeneity among countries. Such calculation methods lead to excessive trade freedom, so the test of the empirical results is not convincing. The calculation of multilateral resistance in this study was based on Head [42] and Novy [45]. The perspective of this method is that the resistance that trading partners encounter when conducting international trade is larger than that of domestic trade. The greater the resistance, the easier it is to conduct domestic trade. Therefore, for an exporting country such as China, both the IM and the EM of the export products will have a negative impact. In addition, the negative impact on the IM is much greater, which indicates that multilateral resistance affects export trade by limiting the IM.

Regarding the impact of SHOCK, in column (2) of Tables 5 and 6, it can be seen that it has a significantly greater negative impact on the IM, which is in line with the expectation, while it has a positive effect on the EM, which is similar to the findings of Sang and Wang [44]. Qian and Xiong [48] and Bernard et al. [55] also found the same result. The emergence of an economic crisis will definitely have an impact on various countries, causing economic weakness and reducing exports [56–58]. Since external shocks frequently constitute force majeure events, this study also included the impact of COVID-19. Judging from the economic situation in the past three years, although China’s economy is gradually recovering, it has not yet reached the level it was at before the pandemic. In addition, China’s exports are dominated by the IM. Therefore, when an external shock occurs, the greater impact on the IM is inevitable.

## 6. Conclusion

In this study, using the binary marginal model, the trade volume of China’s shellfish products exported to the United States, Canada, Japan, and South Korea (the four countries that have implemented the most technical trade barriers to China’s shellfish exports) for 18 years from 2003 to 2020 was examined; the factors affecting shellfish exports were decomposed into the IM and the EM, and the impact of TBT on shellfish exports was deeply analyzed. The main conclusions are as follows. Firstly, the TBT have a significant role in promoting the export of shellfish and aquatic products from China and are mainly driven by the EM. However, with the introduction of external shocks, the TBT restrict the IM to a certain extent. Secondly, China’s export of shellfish and aquatic products is mainly driven by the EM, and the most obvious effect is the fixed trade cost measured by the degree of free trade; that is, the more open the market between countries, the more favorable it is for China to develop new products. Thirdly, in addition to the TBT, the economic scale, productivity level, and external shocks of the trading partner countries also have a restrictive effect on the IM and a driving effect on the EM.

This study puts the following practical implications. Firstly, by employing a binary marginal model to analyze the impact of TBT on China’s shellfish exports, the study provides a nuanced understanding of how TBT affects both the intensive and extensive margins of exports. This enables policymakers to formulate more targeted and effective trade policies to mitigate the adverse effect of TBT on China’s shellfish exports. Secondly, the study highlights the importance of diversifying export markets and product types to reduce the vulnerability of China’s shellfish exports to TBT. By identifying the markets and product types that are less affected by the TBT, exporters can adjust their export strategies to tap into these markets and expand their product portfolio. Furthermore, the study suggests that improving the productivity and quality of shellfish products can enhance their competitiveness in international markets, thereby promoting the expansion margin of exports. This implies that investments in R&D, as well as in improving production processes and quality control, can yield significant returns for exporters. Lastly, the study underscores the role of economic freedom and trade facilitation in promoting trade between countries. By improving the trade environment and reducing trade costs, policymakers can create a more conducive atmosphere for international trade, which in turn can boost China’s shellfish exports.

The study has some limitations. Firstly, the data used in the study may not be fully representative of all shellfish and aquatic products exports from China. The study focuses on a specific subset of exports, which may limit the generalizability of the findings to the broader industry. Secondly, the study uses notified quantities of products as a proxy to reflect the degree of impact of TBT on shellfish exports. While this method provides a quantitative measure of the impact of TBT, it may not capture all aspects of the impact, such as changes in consumer preferences or market access restrictions. Lastly, the study does not consider the potential interactions between TBT and other trade policies, such as tariffs or quotas. These interactions may affect the overall impact of TBT on China’s shellfish exports, and thus should be considered in future research.

## Supporting information

S1 Data Data. (XLSX)

## References

[1] Tan L, Wu C, Qu Y. Measuring the International Trade Competitiveness of China’s Aquatic Products from 2008–18. Journal of Coastal Research. 2020;106(sp1):157. doi: 10.2112/si106-038.1

[2] Wang H, Mao WF, Jiang DG, Liu SJ, Zhang L. Cumulative Risk Assessment of Exposure to Heavy Metals through Aquatic Products in China. Biomed Environ Sci. 2021;34(8):606–15. doi: 10.3967/bes2021.084 34474720

[3] Xu X, Yang Z. Does aquatic products trade waste or save water resources? An analysis of virtual water trade. Water Policy. 2022;24(2):305–23. doi: 10.2166/wp.2022.156

[4] Zeng F. Research on the substitution effect of green barriers for tariff barriers. Finance and Trade Economics. 2003;24(6):61–4. doi: 10.19795/j.cnki.cn11-1166/f.2003.06.013

[5] Cha Y, Koo MG. Who Embraces Technical Barriers to Trade? The Case of European REACH Regulations. World Trade Review. 2020;20(1):25–39. doi: 10.1017/s1474745620000130

[6] Horigan V, Simons R, Kavanagh K, Kelly L. A review of qualitative risk assessment in animal health: Suggestions for best practice. Front Vet Sci. 2023;10. doi: 10.3389/fvets.2023.1102131 PMC9941190 36825234

[7] Islam M. The Sanitary and Phytosanitary Agreement of the World Trade Organization: Debunking Its Reliance on Scientific Evidence and Reluctance to Endorse Potential Biotechnology Risks. Eur J Risk Regul. 2021;12(3):547–63. doi: 10.1017/err.2021.15

[8] Disdier A-C, Fontagné L, Mimouni M. The Impact of Regulations on Agricultural Trade: Evidence from the SPS and TBT Agreements. American J Agri Economics. 2008;90(2):336–50. doi: 10.1111/j.1467-8276.2007.01127.x

[9] Wu J, Wood J, Oh K-Y, Li Y, Bhuyan MI. Impact of TBT and SPS measures on domestic value-added exports: evidence from the United States. Asia-Pacific Journal of Accounting & Economics. 2022;30(5):1150–64. doi: 10.1080/16081625.2022.2077779

[10] Lingling W, Fuli C, Fenghe Z. The dual margin of Chinese agricultural products export to Japan. Management and Entrepreneurship: Trends of Development. 2021;2(16). doi: 10.26661/2522-1566/2021-1/16-04

[11] Huang WP, Cheng DW. An analysis of the trade barriers caused by the sanitary and phyto-sanitary measures (SPS) in international trade contexts. Journal of Renmin University of China. 2001;15(3):54–60.

[12] Crivelli P, Groeschl J. The Impact of Sanitary and Phytosanitary Measures on Market Entry and Trade Flows. World Economy. 2015;39(3):444–73. doi: 10.1111/twec.12283

[13] Zhang Y, Zhu J. Technical barriers to trade and China’s agricultural exports-based on the perspective of specific trade concerns. World Agriculture. 2020;42(9):4–12. doi: 10.13856/j.cn11-1097/s.2020.09.001

[14] Bao XH, Yan XJ. Estimation of binary margin of China’s agricultural export and impact of SPS measures. Journal of International Trade. 2014;40(6):33–41. doi: 10.13510/j.cnki.jit.2014.06.004

[15] Maskus KE, Otsuki T, Wilson JS. The Cost of Compliance with Product Standards for Firms in Developing Countries: An Econometric Study. Policy Research Working Paper. 2005; No. 3590. http://hdl.handle.net/10986/8961

[16] Peng Y. Research on the influence of technical barriers to trade on China’s agricultural product exports-based on empirical research from Japan, the United States, the European Union and South Korea. World Agriculture. 2017;39(4):97–102. doi: 10.13856/j.cn11-1097/s.2017.04.015

[17] Yang W, Wu M. The impact of TBT on dual margin of China’s aquatic exports: an analysis based on the exports to Japan, USA and Korea. Chinese Fisheries Economics. 2017;35(1):67–73.

[18] Guo A. Analysis of the Binary Marginal Impact of SPS Measures on China’s Aquatic Product Exports. Master’s Dissertation. China: Dongbei University of Finance and Economics; 2019.

[19] Qiu B, Das KK, Reed WR. The Effect of Exchange Rates on Chinese Trade: A Dual Margin Approach. Emerging Markets Finance and Trade. 2019;56(15):3709–31. doi: 10.1080/1540496x.2019.1570842

[20] Zhu L. The impact of non-tariff barriers on the dual margin of China’s agricultural exports. World Agriculture. 2017;39(10):140–7. doi: 10.13856/j.cn11-1097/s.2017.10.023

[21] Wang H, Wu Y, Zhu N. Spatio-temporal heterogeneity of China’s import and export trade, factors influencing it, and its implications for developing countries’ trade. PLoS One. 2024;19(4):e0300307. doi: 10.1371/journal.pone.0300307 38635850 PMC11025959

[22] Melitz MJ. The Impact of Trade on Intra-Industry Reallocations and Aggregate Industry Productivity. Econometrica. 2003;71(6):1695–725. doi: 10.1111/1468-0262.00467

[23] Helpman E, Melitz M, Rubinstein Y. Estimating Trade Flows: Trading Partners and Trading Volumes*. Quarterly Journal of Economics. 2008;123(2):441–87. doi: 10.1162/qjec.2008.123.2.441

[24] World Trade Report 2012. The trade effects of non-tariff measures and services measures. [accessed on 10 January, 2025]. https://www.wto.org/english/res_e/booksp_e/anrep_e/wtr12-2d_e.pdf

[25] OECD. The Impact of Regulations on Agro-food Trade. 2003. [accessed on 20 January, 2025] https://www.oecd.org/content/dam/oecd/en/publications/reports/2003/12/the-impact-of-regulations-on-agro-food-trade_g1gh3a91/9789264105423-en.pdf

[26] Wang X, Xu Y, Wang L. Growth dynamics and sustainable development of aquatic products export trade of China and Vietnam. Aquac Int. 2023;:1–25. doi: 10.1007/s10499-023-01119-2 37361882 PMC10110481

[27] Wei H, Tu Y, Zhou P. Technical barriers to trade and export performance: Comparing exiting and staying firms. Economic Modelling. 2023;126:106439. doi: 10.1016/j.econmod.2023.106439

[28] Chaney T. Distorted Gravity: The Intensive and Extensive Margins of International Trade. American Economic Review. 2008;98(4):1707–21. doi: 10.1257/aer.98.4.1707

[29] Chen W-C, Bao X. Technical barriers to trade and China’s exports: firm-level evidence. Applied Economics. 2022;55(17):1919–38. doi: 10.1080/00036846.2022.2100869

[30] Sun C, Niu T. International competitiveness of China’s shellfish products. Chinese Agricultural Science Bulletin. 2015;31(20):44–50.

[31] Yang F, Wang Y, Whang U. Impact of technical barriers to trade measures on innovation – evidence from chinese manufacturing firms. Economics of Innovation and New Technology. 2024;34(5):705–23. doi: 10.1080/10438599.2024.2365316

[32] de Almeida FM, da Cruz Vieira W, da Silva OM. SPS and TBT agreements and international agricultural trade: retaliation or cooperation? Agricultural Economics. 2011;43(2):125–32. doi: 10.1111/j.1574-0862.2011.00570.x

[33] Dong YG, Chu X, Zhao XG. Impacts of sanitary and phytosaniary measures on Chinese exports of agricultural products. Statistics & Information Forum. 2013;28(9):68–74.

[34] Bao X, Zhu D. Differential effects of TBT: global evidence and its insights for China. The Journal of World Economy. 2015;38(11):71–89. doi: 10.19985/j.cnki.cassjwe.2015.11.005

[35] Bao X, Zhu Z. Measurement of TBT and its impact on China’s import trade. The Journal of World Economy. 2006;29(7):3–14.

[36] Fontagné L, Orefice G, Piermartini R, Rocha N. Product standards and margins of trade: Firm-level evidence. Journal of International Economics. 2015;97(1):29–44. doi: 10.1016/j.jinteco.2015.04.008

[37] Dong Y. An empirical study on the impacts of SPS measures on China’s aquatic product export—case of malachite green standards on eel product export as an example. China Rural Economy. 2011;27(2):43–51. doi: 10.20077/j.cnki.11-1262/f.2011.02.005

[38] Hummels D, Klenow PJ. The Variety and Quality of a Nation’s Exports. American Economic Review. 2005;95(3):704–23. doi: 10.1257/0002828054201396

[39] Yu C, Shen Z, Li P. Route Optimization of Aquatic Product Transportation Based on an Improved Ant Colony Algorithm. JACIII. 2020;24(4):488–93. doi: 10.20965/jaciii.2020.p0488

[40] Peng S, Zhou Y, Geng X. Binary Marginal Measure of China’s Fruit Exports. Statistics & Decision. 2020;36(15):75–80. doi: 10.13546/j.cnki.tjyjc.2020.15.015

[41] Tian Z, Du Q. The intensive and extensive margins analysis of Chinese cultural goods export. Macroeconomics. 2019;4:130–43. doi: 10.16304/j.cnki.11-3952/f.2019.04.013

[42] Head K, Mayer T. Chapter 59 The empirics of agglomeration and trade. Handbook of Regional and Urban Economics. Elsevier. 2004. p. 2609–69. doi: 10.1016/s1574-0080(04)80016-6

[43] Shao G, Shao C, Li C. A study of the impact of aquatic product trade cost between China and South Korea on bilateral trade. Review of Economy and Management. 2018;34(4):127–37. doi: 10.13962/j.cnki.37-1486/f.2018.04.011

[44] Sang M, Wang L. Study on influencing factors of China’s export growth to emerging market countries—based on the dual margin analysis framework. Journal of Jianghan University (Social Science Edition). 2020;37(3):89–97. doi: 10.16387/j.cnki.42-1867/c.2020.03.010

[45] Novy D. Gravity redux: measuring international trade costs with panel data. Economic Inquiry. 2012;51(1):101–21. doi: 10.1111/j.1465-7295.2011.00439.x

[46] Gnangnon SK. Multilateral trade liberalization and developing countries’ economic exposure to shocks. JES. 2019;46(2):496–515. doi: 10.1108/jes-05-2017-0141

[47] Liu L, Zhou X, Xu J. Does working capital management improve financial performance in China’s agri-food sector during COVID-19? A comparison with the 2008 financial crisis. PLoS ONE. 2024;19(4):e0300217. doi: 10.1371/journal.pone.0300217 PMC10990196 38568957

[48] Qian X, Xiong P. The dual margin of China export growth and its determinants. Economic Research Journal. 2010;45(1):65–79.

[49] Miao M, Liu H, Chen J. Factors affecting fluctuations in China’s aquatic product exports to Japan, the USA, South Korea, Southeast Asia, and the EU. Aquaculture International. 2021; 29(6): 2507–33. doi: 10.1007/s10499-021-00761-y 34421231 PMC8367770

[50] Nie X, Zuo Z, Zhang R, Luo S, Chi Y, Yuan X, et al. New advances in biological preservation technology for aquatic products. NPJ Sci Food. 2025;9(1):15. doi: 10.1038/s41538-025-00372-4 39900935 PMC11790869

[51] Wei X, Zhang M, Chen K, Huang M, Mujumdar AS, Yang C. Intelligent detection and control of quality deterioration of fresh aquatic products in the supply chain: A review. Computers and Electronics in Agriculture. 2024;218:108720. doi: 10.1016/j.compag.2024.108720

[52] Ragab S, Hoseinifar SH, Van Doan H, Rossi W, Davies S, Ashour M, et al. Overview of Aquaculture Artificial Intelligence (AAI) Applications: Enhance Sustainability and Productivity, Reduce Labor Costs, and Increase the Quality of Aquatic Products. Annals of Animal Science. 2025;25(2):441–53. doi: 10.2478/aoas-2024-0075

[53] Anderson JE, van Wincoop E. Gravity with Gravitas: A Solution to the Border Puzzle. American Economic Review. 2003;93(1):170–92. doi: 10.1257/000282803321455214

[54] Kancs D. Trade Growth in a Heterogeneous Firm Model: Evidence from South Eastern Europe. World Economy. 2007;30(7):1139–69. doi: 10.1111/j.1467-9701.2007.01035.x

[55] Bernard AB, Jensen JB, Redding SJ, Schott PK. The Margins of US Trade. American Economic Review. 2009;99(2):487–93. doi: 10.1257/aer.99.2.487

[56] Feiferytė-Skirienė A, Stasiškienė Ž. Measuring economic crises impact transitioning to a circular economy. Environ Dev Sustain. 2023;:1–25. doi: 10.1007/s10668-023-03367-x 37362983 PMC10202746

[57] Liu S, Chang L, Wang L. Demand forecasting of cold-chain logistics of aquatic products in China under the background of the Covid-19 post-epidemic era. PLoS One. 2023;18(11):e0287030. doi: 10.1371/journal.pone.0287030 38033001 PMC10688662

[58] Wen L, Xu J. The impact of leverage, liquidity, and cash flows on the performance of Chinese agricultural listed companies during COVID-19. Custos e Agronegocio On Line. 2023;19(4):133–48.

